# Supplementary material for: The structure of basal body inner junctions from Tetrahymena revealed by electron cryo-tomography
Source: EMBO J. 2025 Feb 24;44(7):1975–2001. doi: 10.1038/s44318-025-00392-6 (PMC11961760; doi:10.1038/s44318-025-00392-6)
Supplement: Supplementary file 2 — Movie EV1 [file 44318_2025_392_MOESM2_ESM.zip › Movie EV1 legend.docx]

**Movie EV1** (related to Figures 2A and EV2A). A 16-nm repeat structure of the A-B inner junction from the proximal region (9.8 Å resolution). A FAP52-like protein is in red, FAP106 is in purple, and Poc1 is in blue.
