# Supplementary material for: The structure of basal body inner junctions from Tetrahymena revealed by electron cryo-tomography
Source: EMBO J. 2025 Feb 24;44(7):1975–2001. doi: 10.1038/s44318-025-00392-6 (PMC11961760; doi:10.1038/s44318-025-00392-6)
Supplement: Supplementary file 3 — Movie EV2 [file 44318_2025_392_MOESM3_ESM.zip › Movie EV2 legend.docx]

**Movie EV2** (related to Figure 3C). Structure changes in the A-B inner junction before the transition from the proximal to the central core region. Red arrowheads indicate the FAP52-like molecules with 16-nm longitudinal spacing. The black arrowhead indicates an unidentified ladder-like protein in the A-B inner junction. The termination of the A-B inner ladder coincides with the transition from the FAP52-like protein at pf B9 to FAP52_core_ at pf B9/B10.
