# Supplementary material for: The structure of basal body inner junctions from Tetrahymena revealed by electron cryo-tomography
Source: EMBO J. 2025 Feb 24;44(7):1975–2001. doi: 10.1038/s44318-025-00392-6 (PMC11961760; doi:10.1038/s44318-025-00392-6)
Supplement: Supplementary file 4 — Movie EV3 [file 44318_2025_392_MOESM4_ESM.zip › Movie EV3 legend.docx]

**Movie EV3** (related to Figure 3E). The averaged structure shows the changes of TMT transitioning from the proximal to the central core region.
