# Supplementary material for: The structure of basal body inner junctions from Tetrahymena revealed by electron cryo-tomography
Source: EMBO J. 2025 Feb 24;44(7):1975–2001. doi: 10.1038/s44318-025-00392-6 (PMC11961760; doi:10.1038/s44318-025-00392-6)
Supplement: Supplementary file 5 — Movie EV4 [file 44318_2025_392_MOESM5_ESM.zip › Movie EV4 legend.docx]

**Movie EV4** (related to Figures 4A and EV4A). A 48-nm repeat structure of the A-B inner junction from the central core region of BB (9.3 Å resolution). The MIPs are highlighted by colors, FAP52 in red, FAP106 in purple, FAP210 in green, FAP45 in dark orange, IJ34 in light yellow, the LRR-motif MIP in navy blue, and Poc1 in blue.
