# Supplementary material for: The structure of basal body inner junctions from Tetrahymena revealed by electron cryo-tomography
Source: EMBO J. 2025 Feb 24;44(7):1975–2001. doi: 10.1038/s44318-025-00392-6 (PMC11961760; doi:10.1038/s44318-025-00392-6)
Supplement: Supplementary file 6 — Movie EV5 [file 44318_2025_392_MOESM6_ESM.zip › Movie EV5 legend.docx]

**Movie EV5** (related to Figures 4B and 4C). A 16-nm repeat structure of the A-B inner junction from the central core region of BB (8.3 Å resolution). The FAP52 is in red, the FAP106 is in purple, the IJ34 is in light yellow, the LRR-motif MIP is in navy blue, and the Poc1 is in blue.
