# Supplementary material for: The structure of basal body inner junctions from Tetrahymena revealed by electron cryo-tomography
Source: EMBO J. 2025 Feb 24;44(7):1975–2001. doi: 10.1038/s44318-025-00392-6 (PMC11961760; doi:10.1038/s44318-025-00392-6)
Supplement: Supplementary file 7 — Movie EV6 [file 44318_2025_392_MOESM7_ESM.zip › Movie EV6 legend.docx]

**Movie EV6** (related to Figure 4C). An LRR-motif MIP (navy blue) in the A-B inner junction in the central core region of BB. It makes potential interactions with pfs B10, A13, and A1 of the TMT wall, as well as Poc1 (blue) and IJ34 (light yellow). The α/β tubulins are colored in pale green and blue.
