# Supplementary material for: The structure of basal body inner junctions from Tetrahymena revealed by electron cryo-tomography
Source: EMBO J. 2025 Feb 24;44(7):1975–2001. doi: 10.1038/s44318-025-00392-6 (PMC11961760; doi:10.1038/s44318-025-00392-6)
Supplement: Supplementary file 8 — Movie EV7 [file 44318_2025_392_MOESM8_ESM.zip › EMBOJ-2024-119050R-Movie_EV7/Movie EV7 legend.docx]

**Movie EV7** (related to Figure 4E). Comparison of the A-B inner junctions between the BB central core region and the axoneme. The two models are overlaid by using pf A1 as a reference. The helices in pf B10 are displayed as cylinders. The Pf B10 (α/β tubulin) in the central core are in light green and light blue. The Pf B10 (α/β tubulin) in the axoneme is in dark green and dark blue.
