# Supplementary material for: The structure of basal body inner junctions from Tetrahymena revealed by electron cryo-tomography
Source: EMBO J. 2025 Feb 24;44(7):1975–2001. doi: 10.1038/s44318-025-00392-6 (PMC11961760; doi:10.1038/s44318-025-00392-6)
Supplement: Supplementary file 9 — Movie EV8 [file 44318_2025_392_MOESM9_ESM.zip › Movie EV8 legend.docx]

**Movie EV8** (related to Figure 5C). Based on the classification of subtomograms from poc1Δ BB, the average of a subset (Class 3) shows its A-B inner junction (IJ) being filled by axonemal protein FAP20 (orange) and PACRG (dark magenta).
