# Supplementary material for: The structure of basal body inner junctions from Tetrahymena revealed by electron cryo-tomography
Source: EMBO J. 2025 Feb 24;44(7):1975–2001. doi: 10.1038/s44318-025-00392-6 (PMC11961760; doi:10.1038/s44318-025-00392-6)
Supplement: Supplementary file 10 — Expanded View Figures [file 44318_2025_392_MOESM10_ESM.pdf]

Expanded View Figures

Figure EV1. Related to Figs. 1, 2, 4, 5, S5. Assessing resolution of subtomogram averages by Fourier shell correlation (FSC).

The structures and their Fourier shell correlations as a function of resolution (1/Å) are reported in Table 2. (**A**) A 16-nm repeat of the A–B inner junction from the proximal region of BB (wild-type). (**B**) A 16-nm repeat of the B–C inner junction from the proximal region of BB (wild-type). (**C**) A 48-nm repeat of the A–B inner junction from the central core region of BB (wild-type). (**D**) A 16-nm repeat of the A–B inner junction from the central core region of BB (wild-type). (**E**) A 16-nm repeat of the A–B inner junction from the axoneme (wild-type). (**F**) A 16-nm repeat of the A–B inner junction from a subset (Class 3) of the central core region of poc1Δ BB. (**G**) A 48-nm repeat of the A–B inner junction from a subset (Class 3) of the central core region of poc1Δ BB.

 © The Author(s)

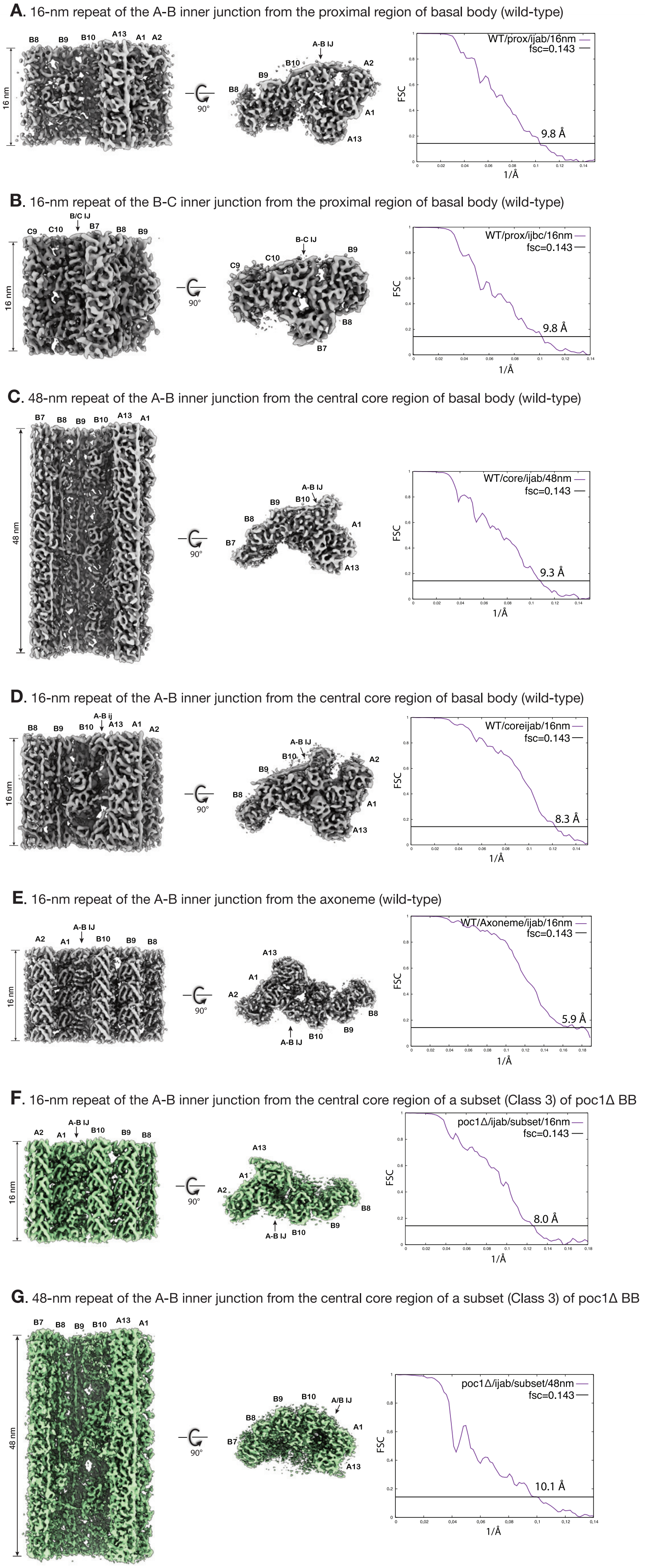
A. 16-nm repeat of the A-B inner junction from the proximal region of basal body (wild-type)
B8 B9 B10 A13 A1 A2
16 nm
90°
A-B IJ
WT/prox/ijab/16nm
fsc=0.143
9.8 Å
FSC
1/Å
B. 16-nm repeat of the B-C inner junction from the proximal region of basal body (wild-type)
B/C IJ
C9 C10 B7 B8 B9
16 nm
90°
B-C IJ
WT/prox/ijbc/16nm
fsc=0.143
9.8 Å
FSC
1/Å
C. 48-nm repeat of the A-B inner junction from the central core region of basal body (wild-type)
B7 B8 B9 B10 A13 A1
48 nm
90°
A-B IJ
WT/core/ijab/48nm
fsc=0.143
9.3 Å
FSC
1/Å
D. 16-nm repeat of the A-B inner junction from the central core region of basal body (wild-type)
A-B ij
B8 B9 B10 A13 A1 A2
16 nm
90°
A-B IJ
WT/coreijab/16nm
fsc=0.143
8.3 Å
FSC
1/Å
E. 16-nm repeat of the A-B inner junction from the axoneme (wild-type)
A-B IJ
A2 A1 B10 B9 B8
16 nm
90°
A13
A-B IJ
WT/Axoneme/ijab/16nm
fsc=0.143
5.9 Å
FSC
1/Å
F. 16-nm repeat of the A-B inner junction from the central core region of a subset (Class 3) of poc1Δ BB
A-B IJ
A2 A1 B10 B9 B8
16 nm
90°
A13
A-B IJ
poc1Δ/ijab/subset/16nm
fsc=0.143
8.0 Å
FSC
1/Å
G. 48-nm repeat of the A-B inner junction from the central core region of a subset (Class 3) of poc1Δ BB
B7 B8 B9 B10 A13 A1
48 nm
90°
A/B IJ
poc1Δ/ijab/subset/48nm
fsc=0.143
10.1 Å
FSC
1/Å

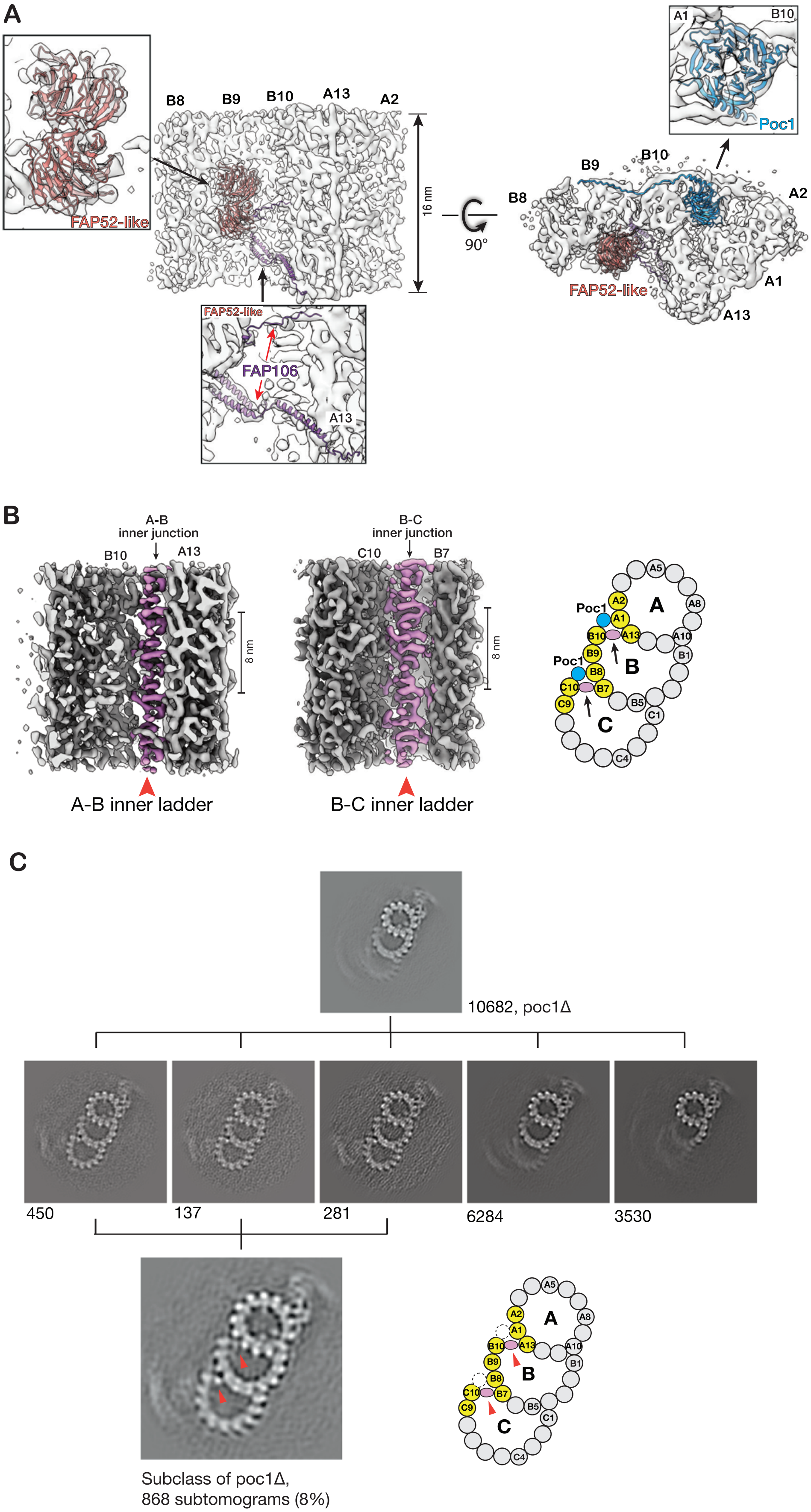

A
FAP52-like
B8
B9
B10
A13
A2
16 nm
90°
A1
B10
Poc1
B9
B10
B8
A2
FAP52-like
A1
A13
FAP52-like
FAP106
A13
B
A-B
inner junction
B10
A13
8 nm
A-B inner ladder
B-C
inner junction
C10
B7
8 nm
B-C inner ladder
Poc1
Poc1
A
B
C
C
10682, poc1Δ
450
137
281
6284
3530
Subclass of poc1Δ,
868 subtomograms (8%)

 © The Author(s)

Figure EV2. Related to Fig. 2. The inner junctions in the proximal region.

(**A**) Fitting the atomic models of FAP52, FAP106 and Poc1 into the 16-nm repeat averaged density map from the proximal region of BB. The FAP52 and FAP106 models are based on the axoneme structure (PDB: 8G2Z). The Poc1 model is from the AlphaFold2 database. (**B**) The maps of the A–B and B–C inner junctions in the proximal region of BB. The two unidentified proteins, namely the A–B inner ladder and B–C inner ladder crosslinking pfs A13-B10 or B7-C10, respectively, are highlighted in light pink and indicated by red arrowheads. On the right, a schematic diagram shows the location of the above two maps in the TMT. Black arrows indicate the viewing directions. (**C**) 3D Classification of the subtomograms from the proximal region of poc1Δ TMT identified a small fraction of the dataset (8%) having complete TMT, where the two unidentified proteins, the A–B inner ladder and B–C inner ladder indicated by red arrowheads, remain in the inner junctions. The number of subtomograms in each class is shown. The dashed circles in the cartoon indicate the location of Poc1 in the wild-type.

© The Author(s)

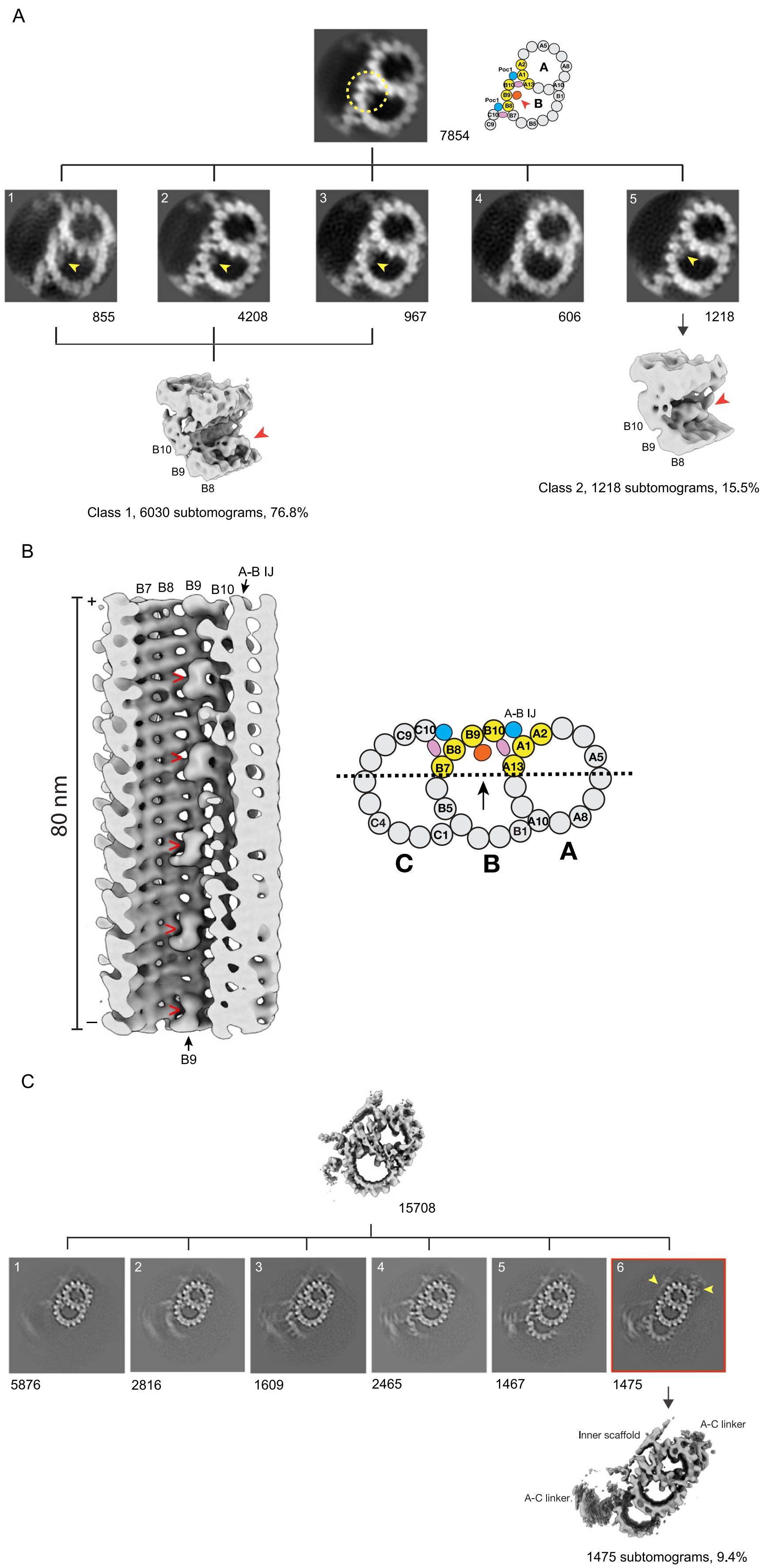
A
7854
A
B
Poc1
855
4208
967
606
1218
B10
B9
B8
Class 1, 6030 subtomograms, 76.8%
Class 2, 1218 subtomograms, 15.5%
B
A-B IJ
B7 B8 B9 B10
80 nm
B9
C9 C10 B9 B10 A2 A1 B8 A13 B7 A5 B5 C4 C1 B1 A10 A8
C B A
C
15708
5876
2816
1609
2465
1467
1475
Inner scaffold
A-C linker
A-C linker
1475 subtomograms, 9.4%

 © The Author(s)

Figure EV3. Related to Fig. 3.

(**A**) Focused 3D classification on the subtomograms from the proximal region of the BB. A yellow dashed circle indicates the focused area centered on the inner junction. Yellow or red arrowheads indicate the locations of FAP52 in the class averages. (**B**) A Longitudinal cross-section of the A–B inner junction showing the FAP52, indicated by red arrowheads, shifts binding from pf B9 to pf 9/10. A schematic illustration of the TMT is on the right. A dashed line and an arrow indicate the cross-section and the viewing direction of the structure on the left. A red dot represents FAP52. (**C**) Focused 3D classification on the subtomograms from the central core region of the BB. The Class 6 is highlighted with a red frame. In this class, the A–C linker and the inner scaffold are indicated by yellow arrowheads. The number of subtomograms in each group is provided.

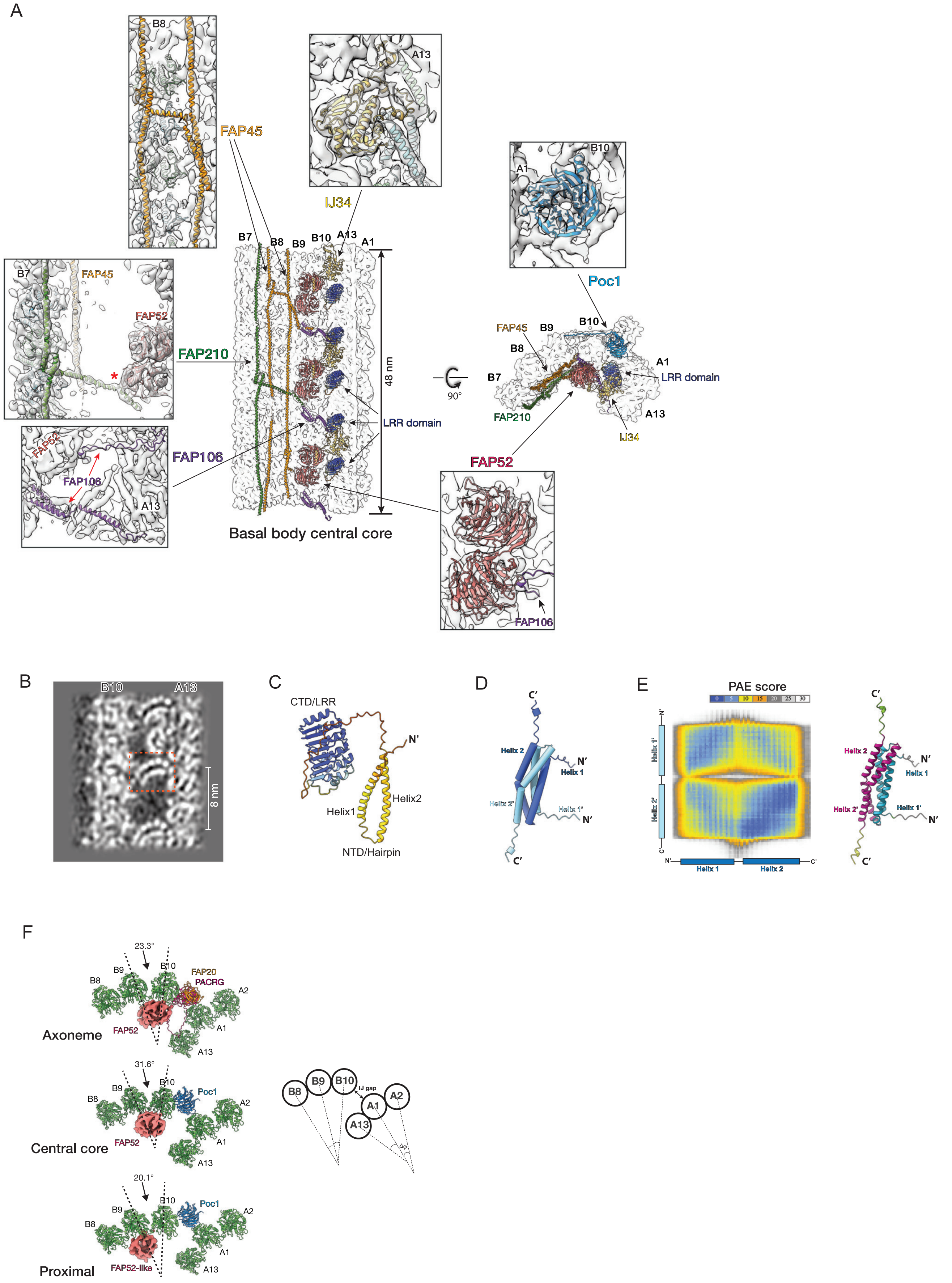
A
B8
FAP45
A13
IJ34
B10
A1
Poc1
B7
B8
B9
B10
A13
A1
FAP45
FAP52
FAP210
48 nm
90°
FAP45
B9
B10
B8
A1
B7
LRR domain
FAP210
A13
IJ34
LRR domain
FAP52
FAP106
FAP106
A13
Basal body central core
FAP52
FAP106
B
B10
A13
8 nm
C
CTD/LRR
N'
Helix2
Helix1
NTD/Hairpin
D
C'
Helix 2
N'
Helix 1
Helix 2'
Helix 1'
N'
C'
E
PAE score
0 5 10 15 20 25 30
Helix 1'
Helix 2'
Helix 1
Helix 2
C'
Helix 2
N'
Helix 1
Helix 2'
Helix 1'
N'
C'
F
23.3°
B9
B8
B10
FAP20
PACRG
A2
Axoneme
FAP52
A1
A13
31.6°
B9
B8
B10
Poc1
A2
Central core
FAP52
A1
A13
20.1°
B9
B8
B10
Poc1
A2
Proximal
FAP52-like
A1
A13
B8
B9
B10
IJ gap
A2
A1
A13

 © The Author(s)

Figure EV4. Related to Fig. 4.

(**A**) Fitting the atomic models of MIPs found in the central core region of BB into the averaged density map. Each inset shows the fitting of a MIP model into its local density and its surroundings. In the "FAP210" inset panel, a red asterisk indicates a potential interaction between FAP210 and FAP52 that has been observed previously in the axoneme structure. In the "FAP52" inset panel, a black arrow indicates a potential interaction between FAP52 and FAP106. The α/β tubulins are colored in pale green and blue in the background. The 16-nm repeat map from the central core region is used for fitting of FAP52. FAP106, IJ34, and Poc1 models, as these MIPs have 16-nm or 8-nm (Poc1) periodicity. (**B**) A cross-section slice of the density map shows an LRR motif highlighted in a red dashed line square. (**C**) An AlphaFold2 predicted protein structure (UniProt Q22N53) was identified previously in the BB proteome. The protein is composed of a N-terminal α-helix hairpin (NTD) and a C-terminal LRR motif (CTD). The structure is colored based on the prediction confidence score (pLDDT: the predicted local distance difference test). The high confidence is in dark blue, while the low confidence is in yellow or orange. (**D**) An AlphaFold 3 predicted 4-helix bundle formed by dimerizing two NTD/hairpins. Two copies of the NTD hairpin from the LRR-motif MIP (UniProt Q22N53) form an anti-parallel dimer. One monomer is in dark blue (Helix 1 and Helix 2) and the other monomer is in light blue (Helix 1′ and Helix 2′). The dimer forms a right-handed 4-helix bundle. (**E**) The predicted aligned error (PAE) plot provides inter-domain packing confidence scores. The dark and light blue imply the prediction with high confidence, while the gray and white indicate low confidence in the interaction. On the right, a ChimeraX-adapted color scheme is used where the 4-helix bundle is colored based on the PAE potential interaction score. The two N-terminal helices (Helix 1, Helix 1′) are in cyan, and the two C-terminal helices (Helix 2 and Helix 2′) are in magenta, indicating that the interaction between Helix 1 and Helix 1′ (cyan), Helix 2 and Helix 2′ (magenta) are with high confidence. (**F**) Inter-protofilament angle measurement. Left: the angles between pfs B9 and B10 are measured at the three regions, showing the variation of local curvature. The FAP52 and Poc1 or FAP20/PACRG are shown as reference points. Right: a schematic diagram depicts the inter-protofilament angles at the A–B inner junction. More complete measurements are in Table 1.

© The Author(s)

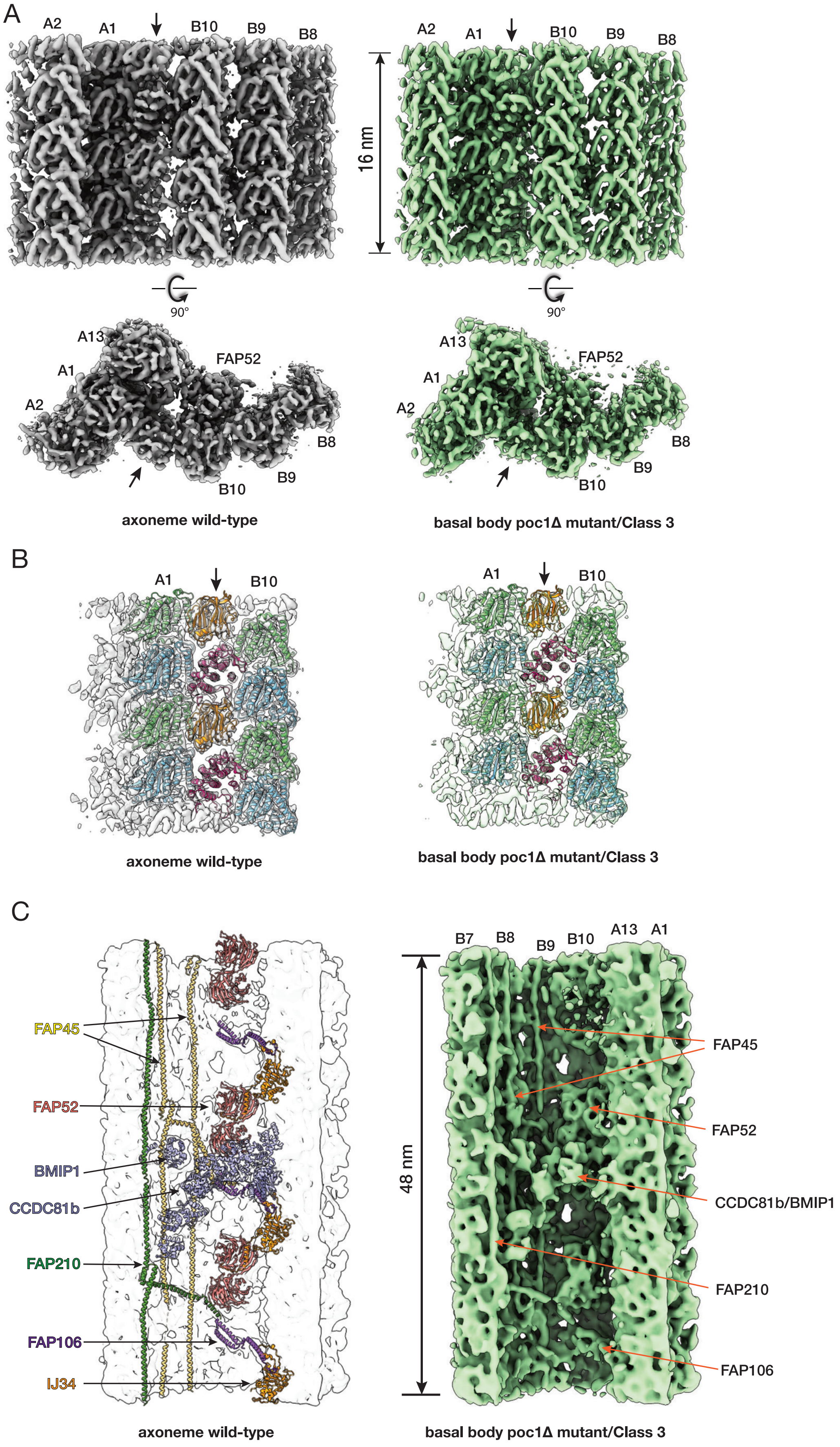
A
A2
A1
B10
B9
B8
16 nm
90°
A13
FAP52
axoneme wild-type
basal body poc1Δ mutant/Class 3
B
C
FAP45
FAP52
BMIP1
CCDC81b
FAP210
FAP106
IJ34
B7
48 nm
CCDC81b/BMIP1

 © The Author(s)

Figure EV5. Related to Fig. 5. Comparing the inner junctions between the wild-type axoneme and the Class 3 from the poc1Δ BBs.

(**A**) Comparing the two structures in two orthogonal views, the wild-type axoneme is in gray, and the poc1Δ BB is in green. (**B**) Fitting the molecular models into the density maps in (**A**). The α/β tubulins are in light green and blue, FAP20 is in orange, and PACRG is in dark magenta. The arrows indicate the A–B inner junctions. (**C**) Comparing the 48-nm repeat from the wild-type axoneme (left) and from a subset of poc1Δ BB (Class 3, on the right in green). The 48-nm repeat average from a subset of poc1Δ BB (Class 3) shows nearly identical structure to the wild-type axoneme inner junction. This is the same subset/class as shown in Fig. 5E, but the longitudinal length is extended to 48 nm here. For clarity, the poc1Δ BB mutant map is low-pass filtered to 12 Å.

© The Author(s)
